# *AMPD1* rs17602729 is associated with physical performance of sprint and power in elite Lithuanian athletes

**DOI:** 10.1186/1471-2156-15-58

**Published:** 2014-05-17

**Authors:** Valentina Ginevičienė, Audronė Jakaitienė, Aidas Pranculis, Kazys Milašius, Linas Tubelis, Algirdas Utkus

**Affiliations:** 1 Department of Human and Medical Genetics, Faculty of Medicine, Vilnius University, Santariškių str. 2, LT-08661 Vilnius, Lithuania; 2 Lithuanian Educological University, Studentų str. 39, LT-08106 Vilnius, Lithuania

**Keywords:** Myoadenylate deaminase, Genetic polymorphism, Genotype, Sprint and power, Endurance

## Abstract

**Background:**

The C34T genetic polymorphism (rs17602729) in the *AMPD1* gene, encoding the skeletal muscle-specific isoform of adenosine monophosphate deaminase (AMPD1), is a common polymorphism among Caucasians that can impair exercise capacity. The aim of the present study was twofold: (1) to determine the C34T *AMPD1* allele/genotype frequency distributions in Lithuanian athletes (n = 204, stratified into three groups: *endurance*, *sprint/power* and *mixed*) and compare them with the allele/genotype frequency distributions in randomly selected healthy Lithuanian non-athletes (n = 260) and (2) to compare common anthropometric measurements and physical performance phenotypes between the three groups of athletes depending on their *AMPD1* genotype.

**Results:**

The results of our study indicate that the frequency of the *AMPD1* TT genotype was 2.4% in the control group, while it was absent in the athlete group. There were significantly more sprint**/**power-orientated athletes with the CC genotype (86.3%) compared with the endurance-orientated athletes (72.9%), mixed athletes (67.1%), and controls (74.2%). We determined that the *AMPD1 C34T* polymorphism is not associated with aerobic muscle performance phenotype (VO_2_max). For CC genotype the short-term explosive muscle power value (based on Vertical Jump test) of athletes from the sprint**/**power group was significantly higher than that of the endurance group athletes (*P* < 0.05). The *AMPD1* CC genotype is associated with anaerobic performance (Vertical Jump).

**Conclusions:**

The *AMPD1* C allele may help athletes to attain elite status in sprint/power-oriented sports, and the T allele is a factor unfavourable for athletics in sprint/power-oriented sports categories. Hence, the *AMPD1* C allele can be regarded as a marker associated with the physical performance of sprint and power. Replications studies are required to confirm this association.

## Background

Physical performance is complex and includes the interaction of genetic and environmental factors. With the rapid development of molecular research in sport, multiple genetic markers associated with physical performance have been discovered (among them are: *ACE, ACTN3, AMPD1, ADRB2, GDF-8, NOS3, PPARGC1A, PPARA, HIF1, MtDNA* markers, etc.) [1-3]. It should be however emphasised that, besides *ACTN3*[4], most sports genetics studies have not yet been replicated in independent samples.

Adenosine monophosphate deaminase (AMPD) is a very important regulator of muscle energy metabolism during exercise [5-7]. AMPD displaces the equilibrium of the myokinase reaction toward ATP production (2 ADP ↔ ATP + AMP) by converting AMP to inosine monophosphate (IMP) [5-9]. Moreover, the AMPD reaction is the initial reaction of the purine nucleotide cycle and plays a central role in the salvage of adenine nucleotides as well as the determination of energy charge [7,8]. Other important functions of the purine nucleotide cycle are the deamination of amino acids and the regulation of the glycolytic pathway by the formation of ammonia and IMP [7-9].

It has been shown that physical activity lowers skeletal muscle AMPD activity. Furthermore, AMPD expression in skeletal muscle is dependent on muscle fibre composition [5,7-9]. In a sprint training study, a decrease in AMPD activity was reported concurrent with an increase in the proportion of fast-twitch (type II) fibres. Therefore, AMPD expression appears to be influenced by the intensity of physical activity [10-12]. The muscle-specific isoform of AMPD (adenosine monophosphate deaminase-1 (AMPD1), also known as myoadenylate deaminase) predominates in all skeletal muscle fibres. The gene encoding this isoform (*AMPD1*) is located on chromosome 1 (1p13). *AMPD1* is mainly expressed in fast-twitch (type II) muscle fibres. Differential *AMPD1* gene expression may contribute to quantitative variations in enzyme activity across muscle groups with different types of fibres [8-10]. The nonsense mutation c.34C > T (C to T transition in nucleotide 34, p.Gln12X, rs17602729) in exon 2 of the *AMPD1* gene converts glutamine codon (CAA) into the premature stop codon (TAA), which results in the early interruption of protein synthesis and appears to be the main cause of AMPD deficiency [1-14]. According to the 1000 Genomes project, the polymorphism of the *AMPD1* was found at a frequency of 11% in European Caucasians (11% in Finland, 14% in England and Spain, and 8% in Italy), 1% in Africans, and 8% in Americans surveyed but was not present in any Asian populations (http://www.1000genomes.org/).

Studies have shown, part of the population who express the mutant *AMPD1* T allele (2% of the Caucasian population are homozygous [TT genotype] and approximately 20% are heterozygous [CT genotype]) are vulnerable to muscular cramps, pain, and premature fatigue during exercises [4,11,15,16]. Tarnopolsky et al. [17] suggested that the mutation of the *AMPD1* might be a harmless genetic variant [17]. Individuals with AMPD1 deficiency (TT genotype) have extremely low skeletal muscle AMPD activity, heterozygous (CT genotype) individuals have intermediate AMPD activity, and homozygous individuals with normal *AMPD1* alleles (CC genotype) have high AMPD activity [9,10]. Moreover, force-generating capacity during repetitive submaximal isometric muscle contractions was shown to be reduced in subjects with AMPD1 deficiency compared with sedentary controls [6]. Colombini et al. [18] have found that in elite endurance athletes the frequency of the *AMPD1* mutant T allele is lower than in controls from the general population [18]. However since no differences in indicators of endurance performance were found between athletes with different genotypes, it was concluded that the *AMPD1* C34T variation does not significantly impair endurance performance [5,18]. Furthermore, two studies reported low frequency of the T allele in a group of top-level Spanish male endurance athletes [5] and Polish rowers [13] compared with controls. Several studies have shown that power-oriented athletes have a significantly lower frequency of the *AMPD1* T allele than controls [7,14]. Varying findings reported by different groups of researchers show that at this point there is no consensus on the actual effect of *AMPD1* polymorphisms on athletic performance.

Therefore the purpose of the present study was twofold. First, we determined the frequency distribution of the *AMPD1* C34T alleles and genotypes in a group of Elite Lithuanian athletes. This group was compared with randomly selected healthy Lithuanian non-athletes. The second aim of this study was to compare common anthropometric measurements and laboratory indexes of muscle strength and endurance performance between the groups of athletes depending on their *AMPD1* genotype.

## Results

The distribution of *AMPD1* C34T polymorphism genotype and allele frequencies in 204 Lithuanian athletes was compared to 260 healthy untrained individuals. Data of genotype and allele distribution analysis is presented in Table 1.

**Table 1:** **Frequency distribution of the C34T *AMPD1 *** **alleles and genotypes**

**Individuals**	**Group size (n)**	**Allele frequencies (%)**		**p-value compared with control**	***AMPD1 *** **C34T genotype frequencies (%)**			**P-value HWE**	**P-value compared with control**
		**C**	**T**		**CC**	**CT**	**TT**		
Endurance group	84	86.9	13.1	0.603	62 (72.9)*	22 (25.9)*	0*	0.167	0.359
Sprint and power group	47	95.7^♦^	4.3^♦^	**0.039**	43 (86.3)*^♣^	4 (11.8)*^♣^	0*^♣^	0.761	**0.010**
Mixed group	73	83.6^♦^	16.4^♦^	0.999	49 (67.1)	24 (32.9)^♣^	0^♣^	0.092	0.216
Total athletes	204	87.7	12.3	0.287	154 (74.2)	50 (24.9)	0	0.050	**0.025**
Control subjects	260	84.0	16.0	-	185 (72.2)	67 (25.5)	8 (2.4)	0.525	-

*χ^2^ = 5.62, d.f. = 2, *P* = 0.021 for *AMPD1* genotype frequencies in *endurance* athletes versus *sprint/power* athletes.

^♣^*χ*^2^ = 9.09, d.f. = 2, *P* = 0.002 for *AMPD1* genotype frequencies in *sprint* ***/*** *power* athletes versus *mixed* athletes.

^♦^*χ*^2^ = 6.32; df = 1; *P* = 0.004 for *AMPD1* alleles frequencies in *sprint* ***/*** *power* athletes versus *mixed* athletes.

Significant mean differences (P < 0.05) are in bold.

Results showed that the *AMPD1* C34T genotype distribution was in line with Hardy-Weinberg equilibrium within all groups (P > 0.05). *AMPD1* genotype frequencies were significantly different between the total athlete group (74.2% CC; 24.9% CT; 0% TT) and the control group (72.2% CC; 25.5% CT; 2.4% TT; P = 0.025) (Table 1). The frequency of homozygous TT genotypes was 2.4% in the control group. The TT genotype was completely absent in the athlete group. There was a significantly lower frequency of the T allele in the sprint/power-orientated athletes (4.3%) compared with the mixed athletes (16.4%) and controls (16.0%). There were significantly more sprint/power-orientated athletes with the *AMPD1* CC genotype (86.3%) compared with the endurance athletes (72.9%), mixed athletes (67.1%), and controls (74.2%) (Table 1). Analysis of the distribution of *AMPD1* alleles between genders in the athlete and control groups revealed no significant differences.

Since the *AMPD1* TT genotype was completely absent in the athlete group, only CT and CC genotypes were used for further analysis. The phenotypical variables of the control group were not measured due to various limitations. The differences between the mean values of the phenotypical variables were analysed with respect to gender and sports groups.

Irrespective of the *AMPD1* genotype, the mean values of anaerobic power parameters — anaerobic alactic muscular power (AAMP), right and left hand grip strength (LGS and RGS) — were significantly higher for males compare to females (P < 0.01) (Table 2). Males carriers of the *AMPD1* CC genotype had significantly lower fat mass than female athletes. Other important anthropometric variables including height, weight, body mass index (BMI), muscle mass; anaerobic power variable —short-term explosive muscle power (STEMP), and aerobic parameter maximum oxygen uptake (VO_2_max) were significantly higher in the male CC genotype athletes (P < 0.02). The latter results confirm a physiological gender difference between phenotypic values. Therefore, as the next step, we compared male and female differences between the *AMPD1* CC and CT genotypes separately. The only significant statistical difference was obtained for STEMP in the subgroup of male athletes. Males carriers of the *AMPD1* CC genotype had higher STEMP compared to the CT genotype male athletes (2010.4 ± 486.2W vs. 1790.2 ± 489.4W; P = 0.018).

**Table 2:** Descriptive summary of phenotypic characteristics of athletes with respect to gender and athlete genotypes

**Phenotype**	**Gender**	***AMPD1 *** **CC genotype**				***AMPD1 *** **CT genotype**				**p-value between genotype**
		**n**	**Mean**	**SD**	**p-value**	**n**	**Mean**	**SD**	**p-value**	
Height (cm)	Total	154	178.3	9.5		50	176.2	9.3		
	Male	123	179.9	9.3	**0.000**	37	177.3	9.7	0.164	0.146
	Female	31	172.2	7.3		13	173.1	7.7		0.736
Weight (kg)	Total	154	73.2	13.4		50	69.4	13.6		
	Male	123	75.4	13.5	**0.000**	37	71.4	14.3	0.082	0.127
	Female	31	64.5	8.6		13	63.8	9.8		0.810
BMI (kg/m^2^)	Total	154	22.74	2.84		50	22.04	3.15		
	Male	123	23.06	3.00	**0.000**	37	22.36	3.45	0.115	0.231
	Female	31	21.47	1.56		13	21.12	1.87		0.526
RGS (kg)	Total	154	46.9	9.6		50	44.9	12.1		
	Male	123	49.5	8.4	**0.000**	37	48.0	11.4	**0.002**	0.469
	Female	31	36.6	6.8		13	36.2	9.7		0.891
LGS (kg)	Total	154	46.0	9.8		50	42.7	11.5		
	Male	123	48.4	8.7	**0.000**	37	45.3	11.1	**0.005**	0.079
	Female	31	36.3	8.0		13	35.2	9.6		0.687
Fat mass (kg)	Total	154	7.98	2.41		50	8.15	2.70		
	Male	123	7.65	2.02	**0.010**	37	7.28	2.01	**0.000**	0.330
	Female	31	9.32	3.26		13	10.62	2.97		0.224
Muscle mass (kg)	Total	154	39.1	8.9		50	36.6	8.8		
	Male	123	40.6	8.5	**0.000**	37	37.7	9.5	0.055	0.072
	Female	31	33.0	7.6		13	33.4	5.3		0.870
STEMP (W)	Total	154	1943.0	486.2		50	1771.8	447.0		
	Male	123	2010.4	489.9	**0.001**	37	1790.2	489.4	0.628	**0.018**
	Female	31	1675.6	370.1		13	1719.3	305.4		0.710
AAMP (W)	Total	154	1204.1	219.3		50	1164.1	218.3		
	Male	123	1253.7	205.5	**0.000**	37	1216.1	225.9	**0.000**	0.342
	Female	31	1007.5	153.3		13	1016.0	97.1		0.854
VO_2max_ (ml/kg/min)	Total	154	61.6	6.0		50	59.8	6.2		
	Male	123	62.2	5.9	**0.019**	37	60.3	6.1	0.321	0.085
	Female	31	59.4	6.2		13	58.3	6.4		0.603

BMI = body mass index. RGS and LGS = right and left hand grip strength. STEMP = short-term explosive muscle power. AARG = anaerobic alactic muscular power. VO_2max_ = maximum oxygen uptake.

Significant mean differences (p < 0.05) between gender groups are in bold.

ANOVA results revealed that irrespective of the *AMPD1* genotype, the sprint/power group athletes had on average higher muscle mass, handgrip strength, STEMP, and AAMP than the mixed group (P < 0.006) (Table 3). Average muscle mass, handgrip strength, and AAMP values were also significantly different between the endurance athletes and the mixed group (P < 0.0001) irrespective of the genotype. For the *AMPD1* CC genotype athletes, average STEMP value of the sprint/power group was significantly higher than the endurance group (P < 0.0001), while for the CT genotype average STEMP value of the endurance group was significantly higher than the mixed group (P < 0.0001) (Table 3). Comparison of the phenotypic characteristics of the sports groups, specifically the *AMPD1* CC and CT genotype athletes, revealed that sprint/power athletes carrying the CC genotype had significantly lower handgrip strength compared with CT genotype athletes (P < 0.05), and mixed athletes carrying the *AMPD1* CC genotype had higher handgrip strength and STEMP compared with the CT genotype athletes (P < 0.03).

**Table 3:** Descriptive summary of phenotypic characteristics of athletes with respect to sport groups and athlete genotypes

**Phenotype**	**Group**	***AMPD1 *** **CC genotype**				***AMPD1 *** **CT genotype**				**p-value between genotype**
		**n**	**Mean**	**SD**	**p-value**	**n**	**Mean**	**SD**	**p-value**	
Height (cm)	Endurance (1)	62	181.4	9.0	p_12_ = 0.157	22	179.1	8.6	p_12_ = 1.000	0.308
	Power (2)	43	177.8	10.0	p_23_ = 0.369	4	179.0	9.3	p_23_ = 0.678	0.826
	MIX (3)	49	174.9	8.3	p_31_ = 0**.001**	24	173.0	9.3	p_31_ = 0.080	0.393
Weight (kg)	Endurance (1)	62	75.3	13.2	p_12_ = 1.000	22	72.9	12.1	p_12_ = 0**.027**	0.446
	Power (2)	43	77.7	14.3	p_23_ = 0**.000**	4	89.9	4.9	p_23_ = 0**.000**	0.100
	MIX (3)	49	66.5	10.0	p_31_ = 0**.001**	24	62.9	11.4	p_31_ = 0**.014**	0.171
BMI. (kg/m^2^)	Endurance (1)	62	22.66	2.34	p_12_ = 0**.003**	22	22.60	2.32	p_12_ = 0**.000**	0.925
	Power (2)	43	24.40	3.37	p_23_ = 0**.000**	4	28.20	2.00	p_23_ = 0**.000**	**0.032**
	MIX (3)	49	21.40	2.14	p_31_ = 0**.037**	24	20.50	2.50	p_31_ = 0**.013**	0.115
RGS (kg)	Endurance (1)	62	47.0	9.9	p_12_ = 0.051	22	49.6	8.6	p_12_ = 0**.023**	0.272
	Power (2)	43	51.3	8.9	p_23_ = 0**.000**	4	63.5	6.6	p_23_ = 0**.000**	**0.011**
	MIX (3)	49	42.8	8.1	p_31_ = 0**.049**	24	37.5	10.0	p_31_ = 0**.000**	**0.018**
LGS (kg)	Endurance (1)	62	46.8	9.5	p_12_ = 0.202	22	46.8	7.3	p_12_ = 0**.010**	0.990
	Power (2)	43	50.2	9.0	p_23_ = 0**.000**	4	61.5	8.4	p_23_ = 0**.000**	**0.020**
	MIX (3)	49	41.2	9.1	p_31_ = 0**.006**	24	35.8	9.9	p_31_ = 0**.000**	**0.021**
Fat mass (kg)	Endurance (1)	62	8.12	2.28	p_12_ = 1.000	22	7.65	1.68	p_12_ = 0**.035**	0.370
	Power (2)	43	7.94	2.51	p_23_ = 1.000	4	11.33	1.91	p_23_ = 0.071	**0.012**
	MIX (3)	49	7.84	2.51	p_31_ = 1.000	24	8.08	3.24	p_31_ = 1.000	0.740
Muscle mass (kg)	Endurance (1)	62	40.3	9.4	p_12_ = 0.246	22	39.7	7.4	p_12_ = 0**.020**	0.785
	Power (2)	43	43.2	8.7	p_23_ = 0**.000**	4	50.2	3.3	p_23_ = 0**.000**	0.119
	MIX (3)	49	34.1	5.6	p_31_ = 0**.000**	24	31.4	6.5	p_31_ = 0**.000**	0.077
STEMP (W)	Endurance (1)	62	1786.6	444.0	p_12_ = 0**.000**	22	1918.2	399.2	p_12_ = 0.080	0.224
	Power (2)	43	2405.9	347.8	p_23_ = 0**.000**	4	2380.5	310.2	p_23_ = 0**.000**	0.889
	MIX (3)	49	1734.8	359.5	p_31_ = 1.000	24	1536.1	353.1	p_31_ = 0**.003**	**0.029**
AAMP (W)	Endurance (1)	62	1224.1	248.3	p_12_ = 0.603	22	1258.9	191.8	p_12_ = 1.000	0.503
	Power (2)	43	1277.8	186.5	p_23_ = 0**.001**	4	1339.0	339.2	p_23_ = 0**.020**	0.562
	MIX (3)	49	1114.3	175.8	p_31_ = 0**.022**	24	1048.0	158.0	p_31_ = 0**.001**	0.122
VO_2max_ (ml/kg/min)	Endurance (1)	62	62.3	5.4	p_12_ = 1.000	22	61.3	4.0	p_12_ = 1.000	0.372
	Power (2)	43	62.1	6.3	p_23_ = 0.567	4	58.4	2.9	p_23_ = 1.000	0.250
	MIX (3)	49	60.4	6.5	p_31_ = 0.298	24	58.6	7.8	p_31_ = 0.409	0.293

BMI = body mass index. RGS and LGS = right and left hand grip strength. STEMP = short-term explosive muscle power. AARG = anaerobic alactic muscular power. VO_2max_ = maximum oxygen uptake.

For each phenotypic characteristic, we report p-values between 12, 23 and 31 groups respectively as indicated in the second column of the table. P-values adjusted for multiple comparisons using the Bonferroni test.

Significant mean differences (p < 0.05) between sports groups and genotype are in bold.

We estimated *post hoc* the statistical power of the Chi-square and ANOVA tests employed in our study by G*power software. We used medium effect size recommended in G*power program and the significance level alpha = 0.05.

Given medium effect size w = 0.3, the power calculation of Chi-square tests for differences in allele frequencies between the athletes and the controls yielded: power = 1.00 for total sample size n = 464 (204 athletes + 260 controls). The empirical power of the Chi-square tests for differences between the allele frequencies in different sport groups was equal to 0.98 and respectively for genotype differences - 0.95.

The empirical power of the ANOVA test for the mean differences of Lithuanian athletes’ phenotypic indexes between three different sport groups for the medium effect size f = 0.25 and the total number of athletes with *AMPD1* CC genotype (n = 154) calculated by G*power was 0.79. The empirical power for *AMPD1* CT genotype group (n = 50) is low and equal to 0.32.

We used G*power to estimate the empirical effect size of the ANOVA test for all phenotypic variables between sports groups separately for *AMPD1* CC and CT genotypes. For *AMPD1* CC and CT genotypes, average empirical effect size was 0.37 and 0.71 respectively. Assuming the empirical effect size as true one, with alpha = 0.05, we obtain that the empirical power is higher than 0.84 and sample size is sufficient to detect large effect size for almost all phenotypic variables except VO_2_max (for CC and CT genotypes), fat mass (CC genotype), height (CT genotype). In order to achieve the power of 0.8 for the latter variables, the sample size should be larger as 1000 which is beyond the possibilities of Lithuanian athletes’ population.

## Discussion

Elite athletic status is a polygenic trait with multiple candidate gene variants playing a certain role, either alone or through complex, gene-gene and gene-environment interactions. In the present study, we investigated the association between *AMPD* C34T polymorphism (rs17602729) and athletic performance in elite Lithuanian athletes group. We chose the *AMPD1* C34T polymorphism due to possible associations with the various aspects of muscle function and capacity that were reported in Introduction. It is a view shared among many researchers that this gene variation has an impact on human physical characteristics [1,5,7,12].

The main findings of the present study were: (i) a significantly lower frequency of the *AMPD1* T allele among the investigated group of elite Lithuanian sprint/power athletes compared to sedentary controls; and (ii) the *AMPD1* CC genotype is associated with anaerobic muscle performance (Vertical Jump). These results are in accordance with previous studies showing that C allele of the *AMPD1* gene is associated with anaerobic performance [5,7,8,12].

The C34T polymorphism in the *AMPD1* gene encoding for the skeletal muscle-specific isoform of AMP deaminase (AMPD1) is a common variation among Caucasians that can impair exercise capacity [3]. Approximately 2% of the general Caucasian population who are homozygous for *AMPD1* rare T alleles (TT genotype) exhibit a skeletal muscle AMPD1 deficiency [7,9].

Results of this study show that the frequency of TT homozygotes in the control group was 2.4%, but none were found among the athletes. We determined the frequency of the T allele to be 16.0% in the control group (in line with other European Caucasian populations) and only 4.3% in sprint/power athletes (P = 0.039). There were significantly more sprint/power athletes with the *AMPD1* CC genotype (86.3%) compared with the endurance athletes (72.9%), mixed athletes (67.1%), and controls (74.2%) (P < 0.025). These results support the association of the C allele with sprint/power performance. Our data on the predominance of the *AMPD1* C allele and CC genotype in sprint/power athletes was in line with the results of other studies on the association of *AMPD1* C34T and anaerobic muscle performance [5,7,8].

Cieszczyk et al. [14] and Fedotovskaya et al. [7] demonstrated that power-oriented athletes had a significantly lower frequency of the *AMPD1* T allele than the controls [5,12].

The potential favourable effect of the *AMPD1* wild-type alleles (CC genotype carriers) on elite sprint power athletic status was additionally supported by the finding that the sprint/power elite athletes with *AMPD1* CC genotype achieved better results in nearly all sprint and power measurements. However, it must be emphasized that no Lithuanian athlete had the TT genotype. Reasons for this striking finding are unclear. We consider that the results of our study are overall sound, as all of the following criteria were met the primary validity criteria for association study [19]: cases (athletes) clearly presented the main study phenotype (i.e. being an elite athlete); genetic assessment were accurate and unbiased; participants within groups were ethnically-matched; and genotype distributions were in HWE in the control (general Lithuanian population) and athletes' groups.

Our study has limitations. Our group of elite athletes was somewhat small, especially after the stratification according to the sports’ category. However considering that there is a limited number of elite athletes in Lithuania, we were unable to recruit additional individuals for this study.

We investigated phenotypes that are related to physical performance to begin creating a chain of evidence linking *AMPD1* C34T to success in sports. Because the *AMPD1* TT genotype was absent in the group of athletes, only CT (n = 50) and CC (n = 154) genotyped athletes were used for the analysis. Aerobic capacity was determined using maximum oxygen uptake (VO_2_max), which is widely accepted as the single best measure of cardiovascular fitness and maximal aerobic power. Instantaneous or explosive power in the lower extremities was measured by a vertical jump test and stair-climbing test (short-term power is the work performed over a brief period of time using maximal effort). Maximal isometric power of the forearm muscles was measured using handgrip strength. These are very rapid movements that are dependent on anaerobic energy use in the muscles and primarily reliant on creatine phosphate. Strength and anaerobic power tests are performance tests most indicative of muscle properties and have been proved to have a significant genetic component [20]. The AMPD1 skeletal enzyme is a very important regulator of muscle energy metabolism during exercise. AMPD1 is activated during short-term, high-intensity exercise, when the rate of ATP use exceeds the cell’s potential to resynthesize it [13]. Previous studies have shown that individuals with the *AMPD1* TT genotype exhibit low AMPD1 activity and a faster accumulation of blood lactate during early recovery from a 30-s sprint exercise [8,9]. Fischer et al. [9] revealed a faster power decrease in TT genotype carriers during the 30-s Wingate cycling test [7]. In a study done by Rico-Sanz et al. [6], individuals with the *AMPD1* TT genotype had diminished exercise capacity and cardiorespiratory responses to exercise in a sedentary state. Furthermore, the training response of ventilatory phenotypes during maximal exercise was lower in *AMPD1* TT genotype carriers [4].

Our results demonstrated that the sprint/power athletes with *AMPD1* wild-type alleles (CC genotype carriers) achieved better results in nearly all sprint and power measurements. *AMPD1* CC genotyped athletes’ from the sprint/power group had an average STEMP value (based on the vertical jump test) significantly higher than endurance group athletes with the same genotype (P < 0.05). Interestingly, we found that athletes in the mixed group (utilising mixed anaerobic and aerobic energy production) carrying the *AMPD1* CC genotype had higher handgrip strength and STEMP than the CT genotype athletes in the same group (P < 0.05).

Our results confirm physiological gender-dependant differences between phenotypic values. *AMPD1* wild-type C allele homozygous males had higher STEMP than the CT genotype male athletes (P < 0.05). Indeed, gender is another factor affecting skeletal muscle AMPD1 activity. Norman (1998) revealed that on average 14–18% higher AMPD1 activities are found in the skeletal muscle of males compared with females [6]. This is similar to what has been observed for other enzymes such as lactate dehydrogenase and phosphofructokinase and suggests a coupling between AMPD1 activity and glycolytic capacity of human skeletal muscles [6]. Our study has confirmed this observation. A statistically significant difference was obtained for the anaerobic STEMP test in the subgroup of CC-genotyped male athletes.

Based on this information, it would appear that the *AMPD1* C allele is favourable for sprint/power performance. This finding is consistent with previous studies that have reported association between the *AMPD1* C allele and status of sprint/power athletes [5,7,8,12].

Our results demonstrated no association for the *AMPD1* C34T polymorphism and aerobic performance phenotype (VO_2_max). Although the latter partly are not significant due to limited sample size, there are other studies those support the same findings. Rico-Sanz et al. found no significant differences between *AMPD1* CC and CT genotype athletes in the VO_2_max values attained by previously sedentary individuals [4].

The results of this study indicate that the effect of the C34T variation of the *AMPD1* gene is essential during intense exercise. Our data indicates that the presence of the *AMPD1* C allele, in conjunction with other environmental and genetic factors, predicts anaerobic capacity.

## Conclusions

Our data suggest that the *AMPD1* C allele may help athletes to attain elite status in sprint/power-oriented sports. Differences in the distribution of *AMPD1* C34T genotypes in the athletes examined and controls and a lower frequency of allele T in sprint/power athletes suggest that allele T is a factor unfavourable for athletics in sprint/power-oriented sports categories. The results indicate the relationship between the *AMPD1* gene C34T variation and muscular activity in an anaerobic mode in humans. Hence, the *AMPD1* C allele can be regarded as one of the markers associated with the performance of sprint and power.

The results provide novel information about the *AMPD1* molecular mechanisms behind physical performance. However, it should be noted that these findings need to be replicated in larger cohorts to confirm the associations. In addition, new physiological and molecular mechanisms behind adaptations to exercise training may be found by studying the genes affecting physical performance. It is important to clarify that although the *AMPD1* gene and the polymorphisms within this gene are important for physical capacity phenotypes, they are not products of a single gene exclusively, because they also heavily depend on composition of muscle fibre, training status, gender, and other environmental and genetic factors.

## Methods

The Lithuanian Bioethics Committee approved the study and written informed consent was obtained from each participant.

### Participants

The study involved 204 athletes (aged 22.0 ± 6.3 years) and 260 controls (healthy unrelated individuals [aged 36.2 ± 7.2 years] from six ethno-linguistic Lithuanian groups). The athletes and control groups were all Caucasians. The 204 elite athletes studied (160 male and 44 female) consisted of Olympic candidates and athletes who had participated in international competitions and had no less than 7 years of experience in their sports categories. The athletes were stratified into three groups according to the duration and distance of the event, in sports disciplines that ranged from endurance-oriented to power-oriented sports. The endurance group (n = 84) included very long (race duration >30 min), long (race duration 5–30 min), and medium (race duration 45 s to 5 min) distance athletes: skiers, road cyclists, biathletes, long-distance runners, modern pentathletes, swimmers, and rowers. The sprint/power group (n = 47) included sprinters and other power athletes with predominantly anaerobic energy production: sprinters, jumpers and throwers. The mixed group (n = 73) comprised athletes whose sports utilized mixed anaerobic and aerobic energy production: wrestlers, tennis players, handball players, and footballers.

### Anthropometric measurements

Body height was measured to the nearest 0.01 m with the subject standing with their back to a wall-mounted stadiometer. Weight was measured to the nearest 0.1 kg with calibrated scales. Body mass index (BMI; in kg/m2) was calculated. Highly trained athletes may have a high BMI because of increased muscularity rather than increased body fat. Total body fat mass (FM, in kg) was determined by measuring the size of the thickest regions of the forearm, humeral area, thigh, and calf and by using a calliper to measure the thickness of the skin, thus determining the amount of subcutaneous fat [21].

### Muscle strength measurements

Anaerobic power was determined with three different tests using the technique recommended by Brown and Weir [21]. Short-term explosive muscle power (STEMP, vertical jump test, in W) was measured by asking the subject to perform a maximal vertical jump (on a contact platform), and the power output expressed per unit of body weight was measured according to Bosco procedures and modifications by Linthorne [22]. Anaerobic alactic muscular power (AAMP, in W) was estimated by a stair-climbing test proposed by Margaria [23-25]. Maximal isometric power of the forearm muscles (handgrip test) was measured according to the procedures described by Mathiowetz et al., with an adjustable mechanical hand dynamometer and expressed in kilograms (kg). For convenience, we denote left and right hand grip strength as LGS and RGS respectively [21,26].

### Measurements of endurance performance

Aerobic capacity was determined by using maximum oxygen consumption (VO_2_max). VO_2_max refers to the maximum amount of oxygen that an individual can utilise during maximal or exhaustive exercise. It is measured as millilitres of oxygen used in 1 minute per kilogram of body weight (ml/kg/min). The gas exchange was measured using a facemask apparatus attached to a continuous, breath-by-breath monitoring system (Oxycon Mobile, Germany). The athletes were evaluated while pedalling on a cycle ergometer (Ergoselect 100 P, Germany) or running on a treadmill (Cosmos Mercury, Germany) according to an incremental procedure until exhaustion. Oxygen uptake, pulmonary ventilation, ventilatory equivalents for oxygen and carbon dioxide, and end-tidal partial pressure of oxygen and carbon dioxide were measured during each test.

All assessments were carried out by trained individuals according to a standardised procedure. Each participant was given two attempts (before and after the preparation period for the most important competitions) and the best attempts were used in the analyses.

### Genotyping

Genomic DNA was extracted from peripheral blood leukocytes by the standard phenol-chloroform extraction method. Genotyping of the *AMPD1* (rs17602729) polymorphism was performed using polymerase chain reaction (PCR). The resulting PCR products were genotyped by restriction fragment length polymorphism analysis. The AMPD1 polymorphism was amplified using PCR forward, 5'-CTTCATACAGCTGAAGAGACA-3' and reverse, 5'-GAATCCAGAAAAGCCATGAGC-3' primers as recommended by Norman et al. [8]. The amplified fragment subsequently underwent digestion by NspI endonuclease (*Thermo Scientific* Fermentas, Lithuania). Digested PCR fragments (216 bp fragments for C allele and 194 bp with 22 bp for T allele) were separated by 2% agarose gel electrophoresis, stained with ethidium bromide, and viewed in UV light.

### Data analysis

Deviation from the Hardy-Weinberg equilibrium was statistically evaluated. Distribution differences between allele and genotype frequencies were tested using chi-square or Fisher’s exact tests. The average differences for each genotype of Lithuanian athletes’ phenotypic indexes were evaluated by using Student’s *t*-test or one-way dispersion analysis (ANOVA). The post-hoc Bonferroni test was applied for multiple comparisons between groups. All phenotypic values (quantitative variables) are presented as the mean ± standard deviation (SD). P-values less than 0.05 were considered statistically significant. The IBM SPSS (v.21) statistical software package was used to obtain the results. We estimated *post hoc* power of the Chi-square and ANOVA tests assuming medium effect size by G*power software [27].

## Abbreviations

AARG: Anaerobic alactic muscular power; ADP: Adenosine diphosphate; AMP: Adenosine monophosphate; AMPD1: Adenosine monophosphate deaminase 1 gene; ANOVA: One-way dispersion analysis; ATP: Adenosine triphosphate; BMI: Body mass index; IMP: Inosine monophosphate; LGS: Left hand grip strength; RGS: Right hand grip strength; SD: Standard deviation; STEMP: Short-term explosive muscle power; VO2max: Maximum oxygen uptake.

## Competing interests

The authors declare they have no competing interests.

## Authors’ contributions

VG, AP performed the genotyping and drafted the manuscript. AJ assisted in the statistical analysis of the data and drafted the manuscript. VG, LT and AU were involved in the recruitment of the study subjects and participated in study design and co-ordination. KM investigated anthropometric measurements and phenotypes that are related to physical performance. All authors read and approved the final manuscript.
